# Role of Antimicrobial Peptides in Immunity of Parasitic Leeches

**DOI:** 10.1134/S0012496623700436

**Published:** 2023-10-13

**Authors:** I. A. Kaygorodova

**Affiliations:** Limnological Institute, Siberian Branch, Russian Acedemy of Sciences, Irkutsk, Russia

**Keywords:** parasitic leeches, immunity, antimicrobial peptides

## Abstract

The review summarizes the current state of knowledge about leech immunity, with emphasis on the special role of antimicrobial peptides (AMPs), and highlights the wide variety of primary AMP structures, which seem to correlate with a variety of life strategies and the ecology of ectoparasites. Antimicrobial proteins and AMPs are a diverse class of natural molecules that are produced in all living organisms in response to an attack by a pathogen and are essential components of the immune system. AMPs can have a wide range of antibiotic activities against foreign and opportunistic bacteria, fungi, and viruses. AMPs play an important role in selection of colonizing bacterial symbionts, thus helping multicellular organisms to cope with certain environmental problems. AMPs are especially important for invertebrates, which lack an adaptive immune system. Although many AMPs are similar in physicochemical properties (a total length from 10 to 100 amino acids, a positive total charge, or a high cysteine content), their immunomodulatory activities are specific for each AMP type.

## INTRODUCTION

Leeches (Hirudinea) belong to segmented worms of the class Clitellata (Annelida). Adults of the class are distinguished by their characteristic citellum, a special organ that produces a reproductive cocoon. More than 720 leech species are known currently and are found in freshwater, marine, and terrestrial ecosystems worldwide; parasitic species account for more than two-thirds of this biodiversity [1, 2]. Leeches emerged during the Cambrian explosion approximately 540 million yiears ago, in a period of rapid expansion of life forms [3–5]. Oligochaeta are thought to be the nearest relative of parasitic leeches. The earliest leech-like worms (Acanthobdellida) mosaically combine the features of the family Lumbriculidae (Oligochaeta) with features of modern leeches in their structure [6–8]. Phylogenetic reconstructions identified the common ancestor of true leeches (Euhirudinea) as a blood-sucking leech that had a proboscis (Rhynchobdellida), while a specific ectocommensal was earlier thought to play the role [9]. The proboscis might have been reduced in carnivorous arhynchobdellid ancestors of leeches during their evolution, allowing them to ingest larger prey. Secondary adaptation to blood sucking might arise in proboscisless leeches of the order Hirudiniformes as a result of their preadaptation to ectoparasitic feeding on the blood. In addition, high homology of genetic sequences was observed between the gnathobdellid (proboscisless) leech *Hirudo medicinalis* and mammals in a transcriptome analysis [10], suggesting coevolution via molecular mimicry of the parasite in a host–parasite system [11].

Various strategies of immune defense developed in leeches during evolution. However, adaptive immunity with an antibody diversification system is absent in leeches, like in other invertebrates. Innate immunity provides a main mechanism that protects leeches from potential pathogens, including water and soil microbes that enter the leech’s body with food or via an injury [12]. Antimicrobial peptides (AMPs) play a special role in the leech immune response, forming an evolutionarily conserved group of protective polypeptides [13].

Lysozyme, which Alexander Fleming discovered in 1922, was the first known AMP. Interest in lysozyme faded once penicillin was found and the golden era of antibiotic discovery began. However, antibiotic-resistant microbial pathogens emerged and started to increase in the early 1960s, and interest in AMPs rekindled. Since that time, AMPs have been observed in virtually all living organisms from archaea to mammals. More than 3500 AMPs are currently described in an AMP database (http://aps.unmc.edu), and that list is far from complete.

An ancient origin and a broad distribution in nature make AMPs a key component of the immune system. AMPs are known to exert a wide spectrum of activities against pathogenic bacteria, fungi, and enveloped viruses and to kill parasites and cancer cells [14]. In addition, AMPs are involved in regulating mutualistic and commensal symbionts and to promote symbiostasis by controlling, determining the composition, and, when necessary, limiting the symbiotic microflora in certain anatomic compartments (the intestine, bacteriomes, and the skin) [15]. Symbionts are known to provide a fast innovation source and thus help animals to adapt to changing environmental factors [16]. Therefore, AMPs can indirectly contribute to the capability of coping with environmental changes in leeches.

Leeches have certain advantages as a model for immunological studies. As an aquatic ectoparasite, a leech has to come in contact through its outer epithelium not only with many microorganisms found in its environment, but also with the host’s outer coating, which has a particular set of ectosymbionts and defense molecules. In a rare feeding period, leeches take maximum advantage of the opportunity and consume twice their body weight [17]. Food digestion is extremely slow, and the host’s blood remains in the parasite’s gut for a long period of time (up to several months in the medicinal leech). The leech digestive system must have a protective mechanism to allow adaptation to the host’s pathogens and to prevent putrefactive processes. Of no less importance is the ability of leeches to regenerate the central nervous system and to restore its function after injury [18]. Anatomic features facilitate the in vitro use of the leech nerve cord. The nerve cord is relatively simple to maintain in culture for several weeks in the absence of peripheral immune system components and blood cells. This makes it possible to focus on the proper immune response that is produced by the leech nervous system [19]. Moreover, clinical studies showed that therapeutic application of leeches does not cause an immune response in the patients [20].

The review considers the specifics of the leech immune system, focusing on the role of AMPs, and highlights the wide variety of primary AMP structures, their functional features and mechanisms of action, as well as the prospects for using AMPs in medicine and other economy sectors.

## LEECH IMMUNE SYSTEM

Like other invertebrates, leeches possess the innate immunity system, while an adaptive (acquired) component is absent in their immune response, having emerged in vertebrates at more recent steps of evolution [21]. Innate immunity plays an important role as a primary barrier to microbial infection. Effectors of innate immunity include pattern recognition receptors, phagocytic cells, proteolytic cascades, and polypeptides with antimicrobial properties. Although innate immunity is a common mechanism, different inner defense strategies can be utilized even in members of the same invertebrate order according to genomic studies [22]. A feature of leeches is that they have both tight associations with symbionts, which may play an important role in inner defense mechanisms, and inevitable contacts with the hosts they infest. The medicinal leech *Hirudo verbana* provides an example. Unusual features of its digestive tract are that its microbial community is simple and that a large amount of the vertebrate blood can be stored for several months [23].

Several immune strategies developed in leeches during their long evolution to protect the body from the microbes that leeches contact in water or soil and the pathogens that find their way in the body through food or wounds [24]. Leeches belong to the group of primitive coelomates and, anatomically, have two compartments that harbor free cells. One is the blood system with hemocytes. This compartment does not appear to be actively associated with immunity. The coelom is the other compartment and contains several coelomocyte populations, which play a certain role in immune defense. Special cell-mediated immunity developed in leeches to target pathogenic microorganisms and includes phagocytosis, encapsulation, and spontaneous coelomocyte cytotoxicity towards allogenic or xenogenic cells. In addition, humoral immunity plays an important role, taking advantage of antimicrobial, hemolytic, and clotting properties of leech bodily fluids.

## CELL-MEDIATED IMMUNE RESPONSE

All annelid worms, including leeches, have effector cells (coelomocytes) that possess spontaneous allogenic and xenogenic cytotoxicities [25]. A coelomocyte contact with a target cell inevitably leads to lysis of the latter [26], similarly to cytotoxic activity of vertebrate natural killer (NK) cells. Like in the NK system, the target specificity is extremely broad, and xenogenic, allogenic, and even syngenic erythrocytes are killed in appropriate conditions. It is possible that cell wall glycoproteins exposed on the surface of target cells are specifically targeted by cytotoxic effectors because certain monosaccharides and disaccharides can block target cell elimination. When coelomocytes are co-cultured with NK-sensitive cell lines, small coelomocytes (SCs) become agitated and extend numerous pseudopodia to bind to and kill the target cells, while large coelomocytes (LCs) aggregate around and encapsulate the lysed targets to form granulomas [27]. SCs are active during recognition and rapidly bind to their targets, while LCs act as phagocytic cells. A group of SCs is responsible for recognition, binding, and killing, while another group of LC-like cells scavenge cell debris. The findings demonstrate that different cell types mediate phagocytosis and cytotoxicity. Leukocytes of annelids are multifunctional, rather than exclusively phagocytic [28], suggesting early divergence of phagocytosis and lysis.

Two different cell types, NK-like and CD8+ cells, were detected in the leech *Glossiphonia complanata* in a study of migration behavior of cells involved in inflammatory reactions [29]. Like in other annelid worms, leech leukocytes phagocytize and encapsulate foreign matter selectively, depending on the size [14]. Encapsulation becomes visually detectable once a parasite is fully coated and isolated in a thick melanotic capsule. When a leech is injured, mass proliferation of lymphocyte-like cells originating from a single cluster (leukopoiesis) is observed in the wound, like in vertebrates.

## HUMORAL IMMUNE RESPONSE

In addition to cell-mediated immunity, lysis and agglutination act as defense mechanisms in invertebrates and are enhanced by coelomic fluid components, such as the antigen-binding protein, cytokines, and antimicrobial substances [30]. Complement-like activity was experimentally demonstrated in annelids in the late 20th century [31]. The coelomic cytolytic factor (CCF) was isolated soon afterwards and proved to have functional similarity to the vertebrate tumor necrosis factor (TNF) [32]. Unlike TNF, the cytolytic activity of CCF is mediated by lysis and is not associated with proteolysis.

In addition to CCF, pore-forming proteins, such as eiseniapore, lysenin, and fetidin, are found in the coelomic fluid of annelids. The presence of sphingolipids is essential for these molecules to bind to and to break the lipid bilayer [33]. A channel complex is utilized by the immune system of annelids to degrade foreign matter. Furthermore, perforin-like proteins form holes in the membranes of target cells [34], facilitating the penetration of lytic molecules, such as lysenins, lysins, fetidins, and hemolysins.

Cytokines are small proteins (5–20 kDa) that play a crucial role in fighting infections and other immune responses [35] by acting as immunomodulatory agents in autocrine, paracrine, and endocrine signal transmission.

As for mitogenic factors, a 60-kDa components with mitogenic activity towards murine splenocytes was identified in the annelid coelomic fluid and termed the coelomic mitogenic factor (CMF) [36]. The same study showed that phospholipase activity is exerted by the CMF-rich annelid coelomic fluid and that a PLA-2-like enzyme is involved in immune responses, including antibacterial mechanisms.

Antigen-binding proteins of annelids consist of two disulfide-bridged polypeptide chains (31 and 33 kDa), which are both involved in forming the antigen-binding site [37].

Protease inhibitors are another class of humoral immunity molecules. A tandem of cystatin B (*Tt-cysb*) and cathepsin L (*Tt-catl*) genes was found in the avian leech *Theromyzon tessulatum* [38]. *Tt-cysb* belongs to the cysteine protease inhibitor family. Its sequence has 54% identity with human cystatin B. *Tt-cysb* is expressed only in one circulating coelomic cell population. Bacterial infection increases the *Tt-cysb* transcript exclusively in these cells.

Among antimicrobial proteins, lysozyme is the best-studied one in annelids [39]. The enzyme cleaves the β-1-4 bonds between N-acetylglucosamine and N-acetylmuramic acid of Gram-positive bacterial cell walls. In addition to lysozyme activity, antibacterial, hemolytic, and hemagglutinating activities appear in the coelomic fluid of the oligochaete *Eisenia fetida* *andrei.* These activities are mainly mediated by two proteins known as fetidins [40].

Leeches have their own lysozyme-like activity: several isofoms of destabilase constitute a protein family characterized by lysozyme activity [41]. In addition, a protein that possess bacteriostatic activity and belongs to the hemerythrin family was found in adipose cells of leeches [24].

In contrast to proteins, AMPs have shorter polypeptide chain (less than 100 amino acid residues) and share the so-called γ-core motif, which is a common signature of all cysteine-stabilized AMPs [42]. Amphipathic and cationic physicochemical properties are characteristic of all AMPs and ensure their biological functions, such as natural antibacterial activity, immune cell chemotaxis, immunomodulation, endotoxin neutralization, and a role in nervous system regeneration [24]. Leech AMPs were isolated from four hematophagous species: the three medicinal leeches *Hirudo medicinalis*, *H. verbena,* and *H. nipponica*, which feed on the blood of mostly warm-blooded animals, and the avian leech *Theromyzon tessulatum*, which infests waterfowl. Lumbricin was the first AMP identified in annelids and was isolated from the oligochaete *Lumbricus rubellus* [43]. A similar AMP was more recently found in leeches [19]. There are currently data on six AMPs observed in leeches: theromacin, theromyzin, peptide B [44], neuromacin, lumbricin [19], and hirudomacin [45].

## AMPs ROLE IN IMMUNE RESPONSE

As mentioned above, AMPs are a component of innate immunity and play an important role in first-line defense against microorganisms, occurring mostly in epithelial tissues of the tegument and the gut. Peptides can be inducible or constitutive, however they are nonspecific, respond before an adaptive immune response is triggered, do not produce immune memory, do not possess catalytic activity, and ensure cell saving by acting as small effector molecules [46].

In addition to direct antimicrobial activity, various immunomodulatory functions are performed by certain AMPs [47].

Some of the most important functions are as follows.

(1) Chemotactic activity. AMPs act directly as chemoattractants that attract immune cells to a site of infection. Acting indirectly, AMPs induce expression of a broad range of chemokines [48].

(2) Antiendotoxin activity. AMPs are capable of suppressing the production of endotoxin-induced proinflammatory mediators, such as TNF-α, by blocking or modulating the Toll-like receptor signaling pathways [49].

(3) Immune cell differentiation. AMPs seem to directly induce cell differentiation and activation, thus integrating elements of innate and acquired immunity [50].

(4) Wound healing and angiogenesis include a regrowth of epithelial layers and formation of new blood vessels. AMPs act directly on epithelial and endothelial cells to trigger reepithelization and angiogenesis. In addition, AMPs act indirectly to stimulate wound healing by exerting their chemotactic effects [51].

In view of their immunomodulatory and antibacterial properties, AMPs are promising candidates for treating infections because they are capable of controlling inflammation in the infections site.

## DIVERSITY OF ANTIMICROBIAL PEPTIDES

### Structure and Properties of Leech AMPs

The discovery of first AMPs in the early 1980s hold great promise for designing new antibiotics [52] and solving the problem of multidrug resistance of pathogenic bacteria [47]. More recent studies showed that AMPs are ubiquitous in living nature and are found in all organism from primitive ones to mammals. Many AMPs proved phylogenetically related, suggesting their evolutionary conservatism [53]. The majority of known AMPs have low molecular weights (2–50 kDa). Main characteristics of AMPs are determined by the facts that, first, AMPs have a positive total charge (usually from +2 to +9) because they harbor many positively charged amino acids, such as lysine and arginine, and, second, AMPs are amphipathic molecules and are stable in both aqueous and hydrophobic solutions [54].

Four types of secondary structures were observed in AMPs: α-helices, β-sheets, combinations of β-sheets and α-helices, and linear or unordered structures [55]. However, most AMPs do not have a definite structure in the free form in solution, but assume their ultimate conformation when interacting with the membrane.

AMPs are synthesized mainly in integumentary tissues, such the skin or intestinal epithelium, the lungs, or red blood cells. AMPs are synthesized 100 times faster than immunoglobulins and at low metabolic costs; moreover, these peptides can accumulate as a reserve within cells to be released when the cells are stimulated by their contacts with pathogens [56]. Thus, AMPs provide a fast nonspecific way to struggle a broad range of microorganisms.

According to the international Antimicrobial Peptide Database (APD), more than 3500 antimicrobial peptides were isolated from various sources in the past 30 years (as of April 2, 2023).

Unicellular organisms produce AMPs for regulation own population size and against other microorganisms competing for environmental space and food resources. A total of 418 AMPs are known now. Of these, the vast majority are bacteriocins (bacterial AMPs), 5 were isolated from archaea; 8, from protists; and 25, from fungi (http://aps.unmc/edu). Their mechanisms of action are diverse and include pore formation, nuclease and peptidoglycanase activities, interference with energy processes, and inhibition of protein synthesis and DNA replication. Gram-negative bacteria produce colicins (25–80 kDa) and microcins (<10 kDa). Bacteriocins produced by Gram-positive bacteria include linear and globular lantibiotics (<5 kDa), peptides without lanthionine (<10 kDa), and high-molecular-weight peptides (>30 kDa).

Plants produce small cysteine-rich AMPs (371 AMPs according to APD), which are found in all organs and accumulate to the greatest extent in the outer layer [57]. Most plant AMPs are 2–10 kDa in molecular weight. These include thionins, defensins, cyclotides, knottin-like APMs, and β-barrelins. The last group is only active against fungi. Thionins, defensins, and cyclotides were found to possess antitumor activity.

Animals are the most important and promising AMP producers (2600 AMPs vs. 371 AMPs of plants or 380 AMPs of bacteria).

In insects, constitutive AMPs accumulate in blood cells and the salivary glands and are secreted into the hemolymph in the presence of microbes. Inducible AMPs are synthesized in microbial infection [58]. Many AMPs varying in structure and activity are currently known in insects. For example, cecropins are active against Gram-negative bacteria, fungi, and viruses and possess insecticidal and antitumor activities. Defensins are active against Gram-positive bacteria. Drosomycin is a potent antifungal peptide. Thanatin exerts antimicrobial activity towards multidrug-resistant clinical isolates of *Enterobacter aerogenes* and *Klebsiella pneumoniae.* Apidaecin acts against Gram-negative bacteria. Mechnikovein is active against Gram-positive bacteria, fungi, and insects. Insecticidal activity was observed for attacin and sarcotoxin. There is also a great diversity of other AMPs.

Aquatic invertebrates were found to possess a wide range of peptides, which possess mostly antibacterial and antifungal activities [59]. Peptides isolated from marine organisms withstand high salt concentrations and, therefore, promise to act successfully at physiological salt concentrations. The set includes AMPs from sea sponges (discodermins, galicilindramides, theonellamides, cyclolitisides, and phoriospongins); jellyfish, corals, and other Cnidaria (aurelins, hydralysins, and stiolysins); mollusks, including cephalopods (mytilus, mytilin, and mycisins); crustaceans (penaedins, callinectins, astacidins, tachyplesin, and tachystatins); starfish (strongyolocins), and annelids (hedistin, perinerin, arenicin, and macins).

**Fig. 1.**
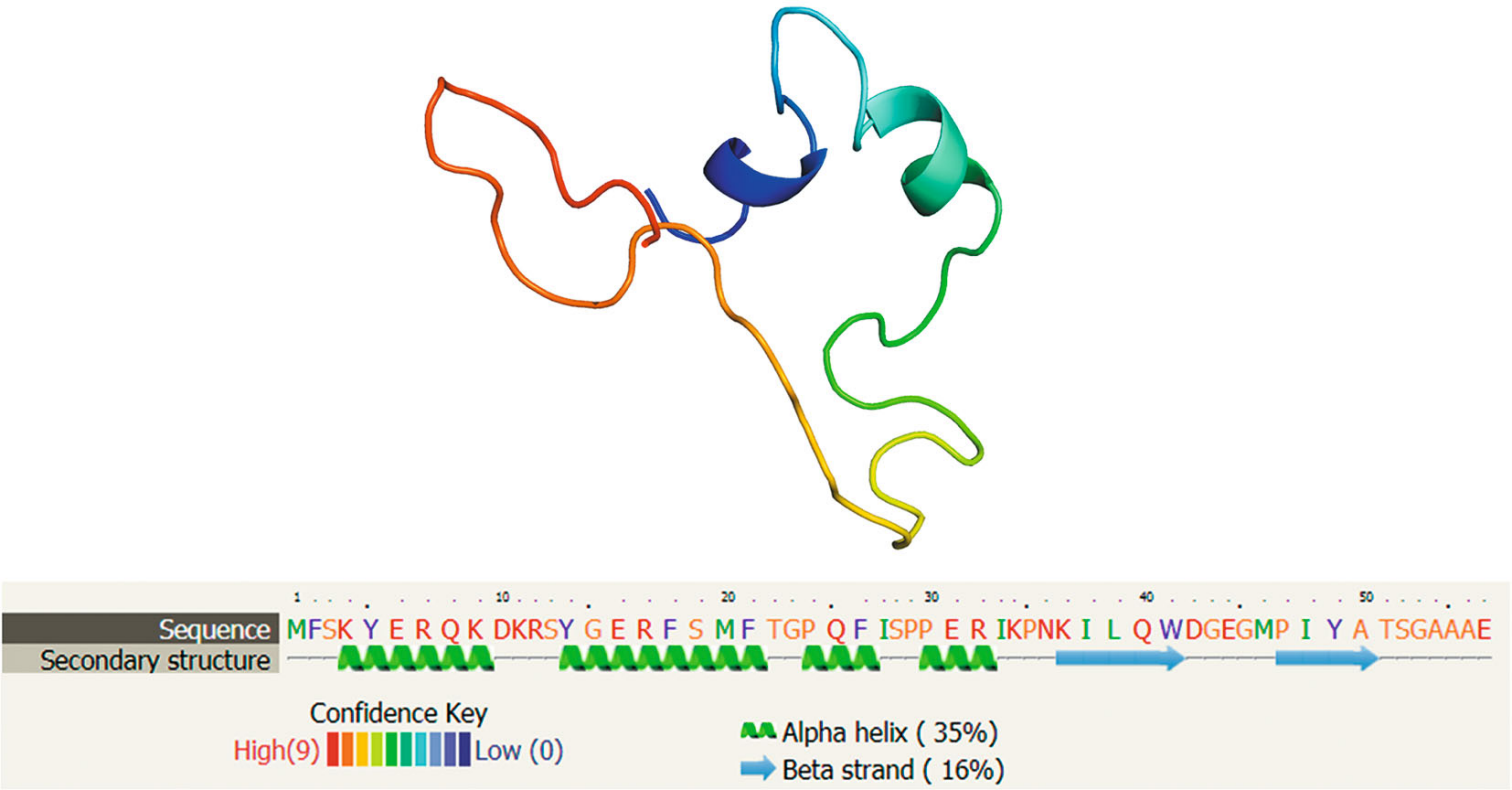
Three-dimensional model of lumbricin of the medicinal leech *Hirudo medicinalis* as reconstructed using Phyre2 [60] and the amino acid sequence deduced from the EU156756 cDNA.

Today, international databases contain data on approximately 20 AMPs that were biochemically isolated from eight freshwater annelid species, including four leeches. Proline-rich lumbricin I, which was isolated from the earthworm *Lumbricus rubellus,* was the first known annelid AMP [43]; its analog was found in leeches more recently [19]. The spatial structure of lumbricin is shown in Fig. 1. A mechanism of action is still unknown for lumbricin-like AMPs. Their antimicrobial activity is relatively low. This makes it possible to assume that protection from pathogens is not a main biological function of lumbricin.

Two different AMPs, theromyzin (an anionic AMP) and theromacin (a cationic AMP) were then discovered in the avian leech *T. tessulatum* [44].

**Fig. 2.**
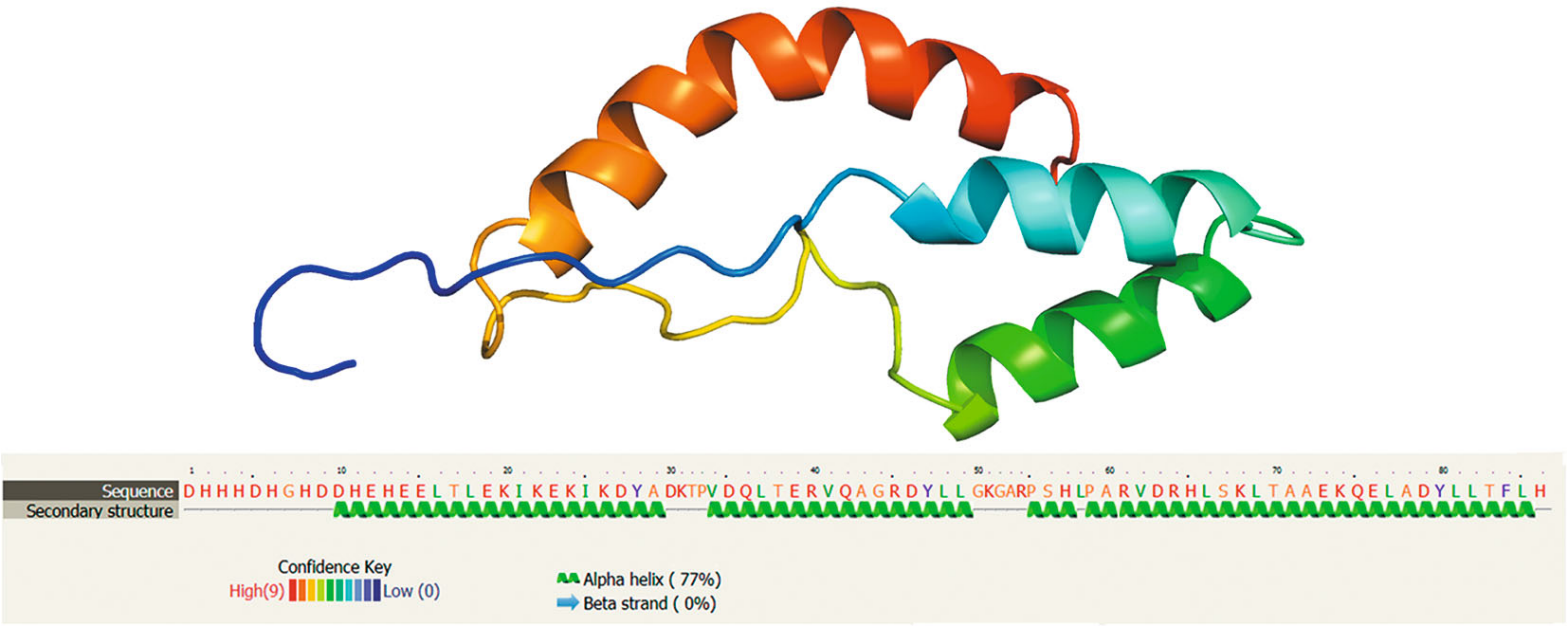
Three-dimensional model of theromyzin the avian leech *Theromyzon tessulatum* as reconstructed using Phyre2 [60] and the amino acid sequence (MedGen UID: 265817).

Theromyzin is a linear polypeptide and consists of 86 amino acid residues. Its spatial structure includes three α-helices (Fig. 2). Theromyzin is the first anionic AMP described in invertebrates. Theromyzin is active against Gram-positive bacteria [44]. Its synthesis is observed exclusively in diffuse tissue, which consists of large adipose cells, which are regularly spread through the leech body and contact the coelome cavities. Theromyzin transcription is activated in response to immune stimuli and increases after feeding or bacterial infection. In addition, theromyzin was immunodetected in the coelomic fluid and intestinal epithelium [44].

**Fig. 3.**
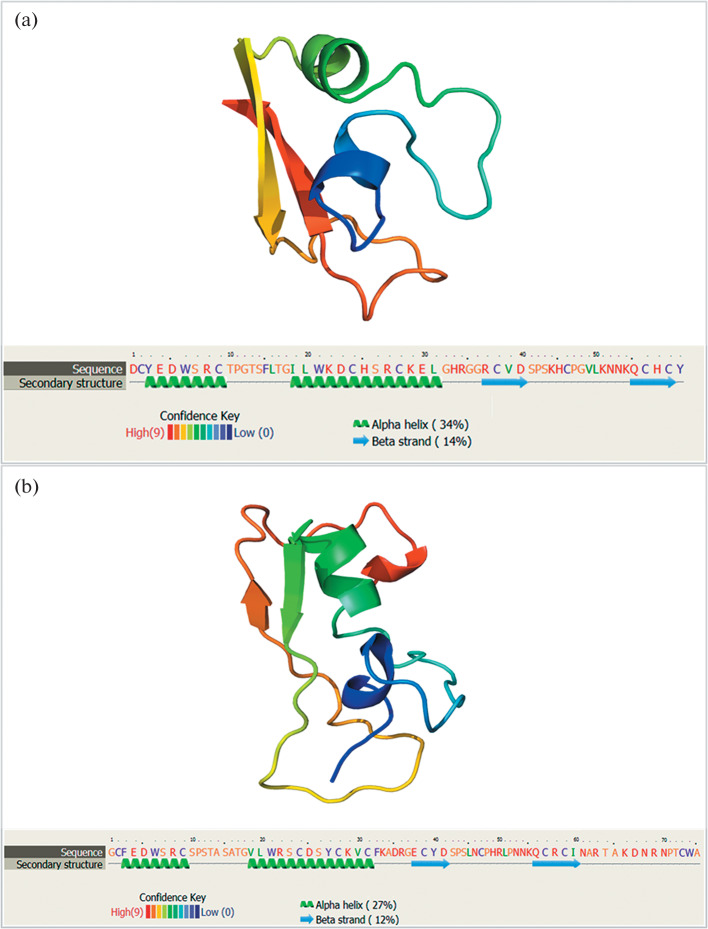
Three-dimensional models of two theromacins as reconstructed using Phyre2 [60]: (a) theromacin of the avian leech *T. tessulatum* (amino acid sequence AP01556) and (b) theromacin of the medicinal leech *H. medicinalis* (amino acid sequence deduced from the EU164975 cDNA).

**Fig. 4.**
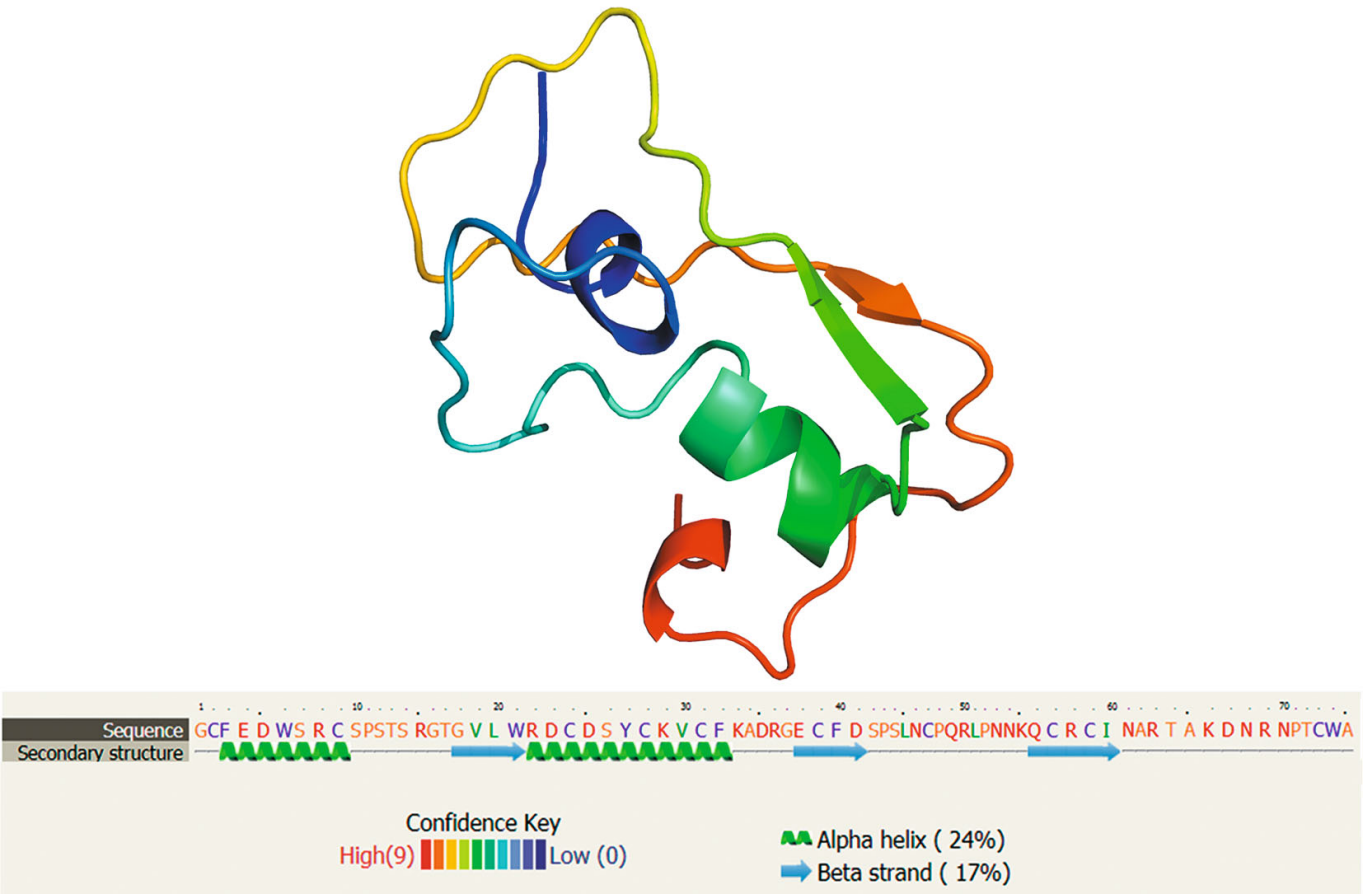
Three-dimensional model of neuromacin of the leech *H. medicinalis* as reconstructed using Phyre2 [60] and the amino acid sequence AP02025.

Theromacin belongs to the family of macins, which were first identified in avian leeches [44]. Macins are a family of cysteine-rich cationic AMPs. Macins have a common motif, CSαβ, which is also characteristic of invertebrate defensins and a family of toxin-like peptides found in scorpions [61]. Members of the peptide family were described in the leeches *T. tessulatum* [44] and *H. medicinalis* [19]. Both leech species are ectoparasitic hematophages of vertebrates. In contrast to defensins, macins have neurotrophic and proliferative activities in addition to their bactericidal effect [19, 61]. AMPs of the macin family are relatively long and complex polypeptide molecules and consist of at least 60 amino acid residues. The macin tertiary structure is organized into a knottin fold according to the arrangement of cysteine bonds, and the molecular surface of a peptide is split into two hydrophobic hemispheres as a result of a strip-like distribution of positive charges [61]. An additional α-helix in the N‑terminal position (Figs. 3, 4) and two flexible loops (are shown as arrows) are conserved structural features of AMPs of the macin family.

Theromacins of the two leech families consist of 75 amino acid residues each [19, 44]. Open cyclic structures of theromacins of the avian and medicinal leeches are shown in Figs. 3a and 3b, respectively. The molecules have similar tertiary structure elements in spite of the 8% difference in amino acid sequence. Compared with other cysteine-rich AMPs of invertebrates, leech theromacins have a different set of cysteine bridges because they harbor more cysteine residues [58]. Theromacins demonstrate antimicrobial activity against Gram-positive bacteria (*Bacillus megaterium* and *Micrococcus luteus*) [61] and low antibacterial activity against Gram-negative proteobacteria (*Escherichia coli*) [44]. Their antimicrobial activity decreases with the increasing salt concentration [61], indicating that theromacins occur exclusively in freshwater leeches.

Neuromacin isolated from the medicinal leech (Fig. 4) consists of 59 amino acid residues and is now the shortest AMP of the macin family. This peptide is similar in general structure to other macins. Neuromacin is produced in neurons and is highly active in relation to Gram-positive bacteria [19]. In contrast to theromacin, neuromacin has four histidine residues in its primary structure, which increase its antimicrobial activity in a weakly alkaline medium. Neuromacin acts as promoter of the regenerative process in the central nervous system (CNS) of the leech in addition to its antibacterial properties. The CNS of annelids, including leeches, is known to differ from the CNS of other animals in being capable of rapid regeneration of neurites and synaptic connections, which restore their normal function after injury. Experimental data showed that bacterial infection stimulates the regenerative process in the medicinal leech and the induction of regeneration of the normal CNS function depends to a great extent on the simultaneous neuroimmune response [19].

Thus, theromacin of *T. tessulatum* [44] and neuromacin and theromacin of *H. medicinalis* [19] perform several functions each, including protection from environmental pathogens, maintenance of gut symbiostasis, and immune defense, and are involved in the regeneration of the nervous system after injury.

**Fig. 5.**
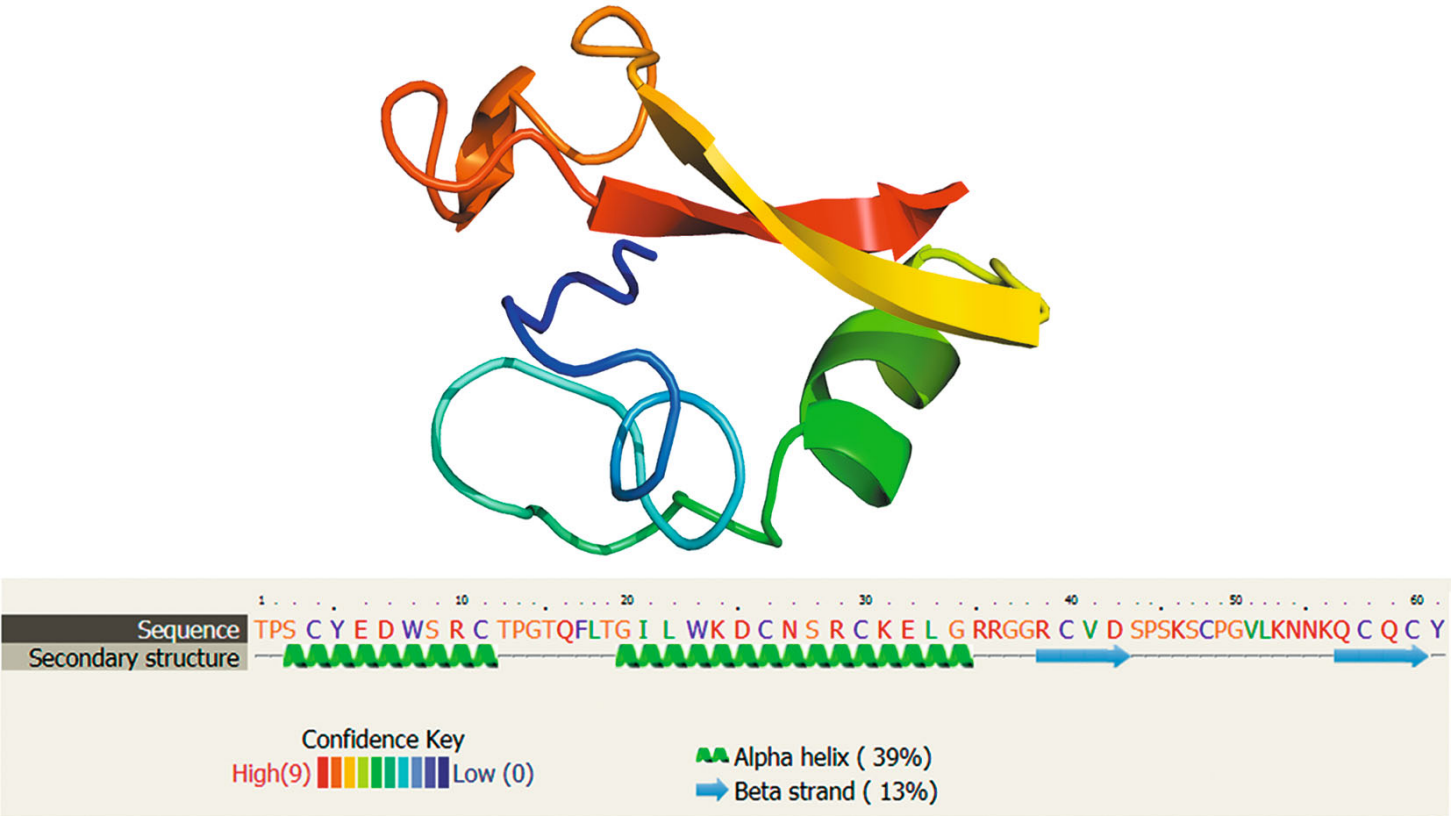
Three-dimensional model of hirudomacin of the leech *H. nipponiсa* as reconstructed using Phyre2 [60] and the amino acid sequence deduced from the MH686152 cDNA.

The new AMP hirudomacin was recently found in the salivary gland of the Asian hematophagous leech *H. nipponica*; its sequence consists of 61 amino acid residues [45]. Hirudomacin has eight cysteine residues, which form four disulfide bridges, and is structurally most similar to members of the macin family. Hydrogen bonds stabilize the hirudomacin conformation with α-helical and β-sheet secondary structures (Fig. 5). The mature polypeptide has antimicrobial activity against a broad range of Gram-positive and Gram-negative bacteria. Hirudomacin synthesis in *H. nipponica* is activated at the feeding stage in the presence of a vertebrate host’s blood.

BRICHOS is another family of cysteine-rich AMPs found in annelids. Arenicin was the first family member and was isolated from the coelomic fluid of the marine polychaete *Arenicola marina* [62]. The BRICHOS domain is now known to occur in precursors of the AMPs alvinellacin, capitellacin [63], and nicomicin [64]. A β-hairpin stabilized by one or two disulfide bridges is a secondary structure characteristic of the majority of peptides of the family [63]. However, there are data that the BRICHOS domain is present as well in precursors of α-helical AMPs, which lack β-hairpins in their structures [64].

Precursor proteins of these AMPs are involved mostly in cell chemotaxis. There is evidence that BRICHOS is involved in complex posttranslational processing of proteins and functions as an intramolecular chaperone domain, which can bind the β-hairpin motifs to prevent their self-aggregation with the formation of amyloid fibrils [63]. The presence of the BRICHOS domain is possibly an evolutionary adaptation that ensures a proper folding of leech AMPs in extreme conditions, such as oxygen deficiency (hypoxia) or thermal stress.

## MECHANISM OF ACTION

A cationic charge and a substantial portion of hydrophobic residues are the main physical properties of AMPs. The former provides for selectivity towards negatively charged microbial plasma membranes vs. zwitterionic membranes of multicellular organisms. The latter property facilitates the interactions with acyl chains of fatty acids [65].

Many linear AMPs are unordered in aqueous solutions, and a membrane environment is necessary for them to assume a stable amphipathic conformation. Impairing the integrity of the bacterial plasma membrane by various means is part of the mechanisms of action of many AMPs. Other antimicrobial mechanisms are also known now and target the key cell processes, such as DNA and protein syntheses, protein folding, enzymatic activities, and synthesis of the cell wall [66, 67]. The cationic properties of AMPs facilitate their interactions with negatively charged moieties of other biomolecules, including outer membrane lipids, nucleic acids, and phosphorylated proteins. Several isoforms or structurally similar AMPs can be produced simultaneously in an infected animal and can act via different mechanisms to produce a synergistic overall effect [68]. In addition, certain AMPs utilize several mechanisms of action, that is, a multiple-hit strategy, which increases their efficiency and helps to evade potential resistance mechanisms.

Thus, AMP mechanisms of action are possible to classify into two categories, membrane disrupting and non-membrane disrupting [69]. The interaction with the plasma membrane via electrical charges is the initial step in either case.

Membrane disruptors account for a vast majority of AMPs. Many of them have α-helical structures, which act directly at the plasma membrane level to alter the cell permeability or to cause cell lysis via pore formation. According to biological activity, two groups of membrane disruptors can be distinguished. These are selective AMPs, which are active towards bacteria and inactive towards animal cells, and nonselective AMPs, which are active towards both bacterial and animal cells.

The mechanism of action of α-helical AMPs includes the following steps [69].

1. Electrostatic interactions determine the AMP binding with membrane phospholipids. AMPs are arranged on the membrane so that their positively charged amino acid residues meet the negatively charged heads of phospholipids. This allows AMPs to preserve their parallel orientation relative to the membrane at a low local peptide concentration. AMPs remain inactive in this state.

2. When the local peptide concentration increases, the peptide tends to assume a perpendicular orientation relative to the membrane. The peptide becomes active in this state and intercalates into the membrane.

3. Finally, peptide intercalation changes the permeability of the lipid bilayer or cell lysis results from the formation of barrel-stave pores or structural alteration of toroidal pores.

Non-membrane disruptors utilize a variety of mechanisms of action, including the binding to nucleic acids (DNA or RNA), interference with nucleic acid synthesis, inhibition of protein synthesis, inhibition of enzymatic activity, inhibition of cell-wall synthesis, cell damage due to intracellular peptide accumulation, flocculation of intracellular components, structural changes in septa, and blockage of certain virulence factors [66, 70]. The effects suppress bacterial motility or reduce bacterial pathogenicity, thus helping to control the infection.

Apart from direct antimicrobial activity, immunomodulatory mechanisms can be utilized by AMPs to protect the leech body [71]. AMPs can exert chemotactic activity to attract immune cells; modulate the leech cell sensitivity to ligands of the Toll-like receptors; stimulate angiogenesis during regeneration of damaged tissue, elimination of inflammation foci, scar formation, and other restorative processes; and modulate expression of anti-inflammatory cytokines or chemokines.

A special role is played in the gut by AMPs, which act as innate regulators [72]. The intestinal epithelium is exposed to numerous pathogens, and endogenous AMPs are essential to act in the gut to prevent foreign invasions and infections. It becomes clear that AMPs determine the composition of the commensal microbiota and help to maintain intestinal homeostasis in leeches and other organisms [15]. AMP expression is strongly controlled with the involvement of pattern recognition receptors, and their distortion is associated with abnormal responses to infection and inflammatory bowel disease.

The macins mechanism of action, as well as other cationic AMPs, includes an increase in membrane permeability in Gram-positive bacteria and pore formation [73]. Due to their structural features and double-amphipathic character (two hydrophobic hemispheres sandwiched by a belt of positive charges), macins promote aggregation of bacteria and then permeabilize the bacterial membrane. Neuromacin and theromacin take part in regeneration of nerve fibers in addition to exerting antibacterial activity [19, 61]. In the medicinal leech *H. medicinalis,* theromacin is released into the blood surrounding the nervous system and neuromacin is procued by nerve cells and accumulates in a damaged site of the central nervous system [74]. In the avian leech *T. tessulatum,* theromacin is expressed in large adipose cells and is released into the coelomic fluid immediately after infection or injury of the central nervous system [44, 61].

## AMP APPLICATIONS

Structural and functional characteristics of AMPs, as well as their properties such as low toxicity to eukaryotic cells, a wide spectrum of activity against various pathogenic microorganisms, and immunomodulatory effects make AMPs valuable therapeutic and prophylactic agents [75]. Although certain limitations still exist, controlling the AMP level may present an important paradigm in treating a great variety of human and animal diseases.

Indirect functions of AMPs, one of which is their participation in the formation of the commensal microbiota composition in animals and humans, are also of interest in the terms of AMP applications in medicine. Destabilization of the microbiome may have adverse health consequences, leading to obesity, recurrent infections, inflammatory bowel disease, and atopic dermatitis. Often, these symptoms are clinically associated with defective antibacterial mechanisms and, in particular, defensin deficiency [71]. In turn, excessive AMP synthesis may cause inflammatory diseases. Certain dermatoses, such as psoriasis and rosacea, are accompanied by increased expression of the AMP cathelicidin [71]. Given their role as modulators of innate immunity, AMPs are potentially suitable for developing immunomodulatory interventions to treat or correct the disorders of the kind.

Interest in AMPs is explained mostly by their prospective use as radically new next-generation anti-infective agents and new methods for more selective control of pathogens. This is certainly of importance for solving universal problems associated with a decrease in the potency of conventional antibiotics due to the increased drug resistance of pathogenic bacteria. The mechanisms utilized by AMPs make the development of bacterial resistance very difficult, opening up a promising future for the use of AMPs as more resistant therapeutic agents. Important advantages of AMPs are that microorganisms lack a mechanism of hereditary transmission of AMP resistance and that the natural microflora is not affected by AMP treatment, in contrast to treatment with conventional antibiotics [47]. Another advantage is their ability to recognize certain types of plasma membranes. This selectivity allows AMPs to differentially recognize normal cells, non-malignant tumor cells, and malignant tumor cells; therefore, AMPs can find application in oncology [69].

AMPs meet many requirements of pharmaceutical industry, agriculture, aquaculture, and food industry. Certain AMPs have already found their application. Bacterial AMPs (bacteriocins) are used to inhibit important animal, human, and plant pathogens, including enterotoxin-producing *E. coli*, resistant staphylococci and enterococci, and *Agrobacterium* and *Brenneria* spp. [76]. For example, the bacterial peptide nisin is broadly used as a safe food preservative and a means to keep roses fresh and to treat fish. Mammalian defensins and cathelicidins proved active against the human immunodeficiency virus (HIV) and other sexually transmitted diseases [66]. Frog skin AMPs exert spermicidal activity and can find application as nonhormonal contraceptives [77]. Transgenic plants that express AMPs come to be used in crop production instead of pesticides, which are toxic to humans and hazardous to the environment [57]. Manipulating the AMP genes in crabs, shrimp, and mussels yields organisms that have higher disease resistance and are better suitable for cultivation [59].

As for leeches, their anticoagulating effect has been known from ancient time, and leech therapy is still in use in both Europe and Asia [20]. Applied interest in leeches is rekindling now. While sucking the blood, hematophagous leeches were found to release not only hirudin, but also other biologically active compounds to prevent blood clotting and decay [45]. Leech peptides have many important advantages, of which the most specific is the capability of overcoming the immune response during regeneration of the nervous system [73]. Benefits of pharmaceutical formulations based on leech AMPs include biochemical compatibility with vertebrate hosts, lack of toxicity towards the commensal microflora, thermal stability, salt resistance, and a broad antimicrobial activity spectrum [78]. Life strategies are highly diverse in leeches (parasitic or nonparasitic, specialists or generalists, endemic or cosmopolitan, extremophilic or normophilic, etc.), but AMPs have been studied only in a limited number of hematophagous species that infest vertebrates. This opens up wide opprtunities to search for new, possibly unique AMPs.
